# Supplementary material for: Economic cost of patients with trisomy 13, 18, and 21 in a tertiary hospital in Thailand
Source: PLoS One. 2023 Nov 16;18(11):e0291918. doi: 10.1371/journal.pone.0291918 (PMC10653468; doi:10.1371/journal.pone.0291918)
Supplement: Attachment — Submitted filename: Comment_PLOS ONE.docx [file pone.0291918.s001.docx]

**Comment**

This study assessed the healthcare use and associated costs for patients with trisomy 13, 18, 21 in Thailand. Overall, the findings of the study benefits to further economic research in Thailand. However, some concerns need to be addressed.

1. The title of the study specified “Economic cost of patients with trisomy 13, 18, 21 in Thailand”. However, only one site with very small sample size (trisomy 13, 18) might not represent the cost for the whole country?
2. The study uses descriptive statistics to provide % cost contribution, costs of outpatient and inpatient. Those costs did not control for other confounders such as age, other co-morbidities, gender, different length of stay. A more robust analysis like GLM regression could provide a more robust result?
3. The data were retrieved via ICD-10-CM. How many years are there for the database?
4. Direct non-medical costs were calculated from standard costing. Why did authors use the standard costing? Need to explain the strength of the standard costing. Does it still reflect direct non-medical costs nowadays?
5. Indirect cost was determined using a human capital approach. The majority of population in this study is children. Is this approach appropriate for those who are not working? In addition, the detail of calculations should be added.
6. Where did 4% (page 6) come from and how did author apply in the formular given?
7. Table 2: what is the unit of annual direct medical cost for OPD and IPD? Is it per visit, per admission, or per patient? Table 1 shows number of visits, and LOS. Authors should classify them by CHD.
8. Drug and medical device are the main component of costs. Do they include drug costs for co-morbidities?
9. Table 6 displays the annual average cost. Indirect costs of each trisomy were equal. Why? Furthermore, indirect cost of patient was equal to that of care giver? Patients were children, but care givers were generally their parents.
10. According to Table 1, age range of patient with trisomy 13 and trisomy 18 was 0-14 years. Table 6. Why is lifetime cost equal to annual total cost for Trisomy 13 and Trisomy 18? Patients can live longer than a year. In addition, how did author calculate lifetime cost? The detail should be added.
11. Authors might display some results using pictures instead of tables.
