# Supplementary material for: Economic cost of patients with trisomy 13, 18, and 21 in a tertiary hospital in Thailand
Source: PLoS One. 2023 Nov 16;18(11):e0291918. doi: 10.1371/journal.pone.0291918 (PMC10653468; doi:10.1371/journal.pone.0291918)
Supplement: Attachment — Submitted filename: Maunscript_PLOS_Reviewed.docx [file pone.0291918.s002.docx]

**Economic Cost of Patients with Trisomy 13, 18, 21 in Thailand**

Preechaya Wongkrajang^1,2^, Jiraphun Jittikoon^3^, Wanvisa Udomsinprasert^3^, Pattarawalai Talungchit^4,6^, Usa Chaikledkaew^5,6^*

^1^Social, Economic and Administrative Pharmacy (SEAP) Graduate Program, Faculty of Pharmacy, Mahidol University

^2^Department of Clinical Pathology, Faculty of Medicine Siriraj Hospital, Mahidol University

^3^Department of Biochemistry, Faculty of Pharmacy, Mahidol University

^4^Department of Obstetrics and Gynecology, Faculty of Medicine Siriraj Hospital, Mahidol University

^5^Social and Administrative Pharmacy Division, Department of Pharmacy, Faculty of Pharmacy, Mahidol University

^6^Mahidol University Health Technology Assessment (MUHTA) Graduate Program, Mahidol University

*Corresponding author

E-mail: [usa.chi@mahidol.ac.th](mailto:usa.chi@mahidol.ac.th)

Orchid Number 0000-0001-9457-9823

**Abstract**

The purpose of this study was to determine direct and indirect costs of patients with trisomy (T) 13, 18, 21 in Thailand. Direct medical costs were obtained from Siriraj Informatics and Data Innovation Center (SiData+), Faculty of Medicine, Siriraj Hospital, and indirect costs were estimated using a human capital approach. About 241 patients with T21 had outpatient care visits and 124 patients received inpatient care. For T13 and T18, five and seven patients were analyzed for outpatient and inpatient cares, respectively. For patients with T13, T18, and T21 receiving outpatient care, total annual mean direct medical costs ranged from 183.2 USD to 655.2 USD. For inpatient care, average yearly direct medical costs varied between 2,507 USD to 14,790.5 USD. The mean and median both increased with age. In outpatient care, costs associated with drugs and medical devices were a major factor for both T13 and T21 patients, whereas laboratory costs were substantial for T18 patients. For inpatient care, costs of drug and medical devices were the greatest for T13 patients, while service fee and operation costs were the highest for T18 and T21 patients, respectively. For outpatient care, adult group with congenital heart disease (CHD) had significantly higher mean annual direct medical costs than those without CHD. However, all patients and children group with CHD receiving inpatient care had significantly higher costs. Patients with T13, T18, and T21 had relative lifetime costs of 30,128 USD, 19,158 USD, and 1,153,629 USD, respectively

**Introduction**

Aneuploidies or chromosomal abnormalities affecting 0.5 to 1.0% of live births may cause a variety of health problems, with trisomy 21, 18, and 13 being the most prevalent [1]. One in every 700–800 live births is affected with trisomy 21 (T21) or Down syndrome, and the average life expectancy of those affected has grown by 50 years in the last century [2]. There are several clinical issues with varying degrees of severity, including poor muscle tone, intellectual and developmental disabilities, heart disease, gastrointestinal defect, hematologic abnormalities, hypothyroidism, and other endocrine abnormalities [3,4]. Trisomy 18 (T18), often known as Edwards syndrome, is the second most frequent kind of trisomy. The incidence of T18 is one in 3,000 to 8,000 live births and the ratio of females to males is 3:1. More than 95% of these fetuses are lost due to spontaneous abortion. Less than 1% of affected infants survive until their first birthday [1,5]. Most affected patients have a particular phenotype, including craniofacial features such as dolichocephaly with a prominent occiput, and clinical abnormalities such as heart disease, ophthalmologic and otolaryngologic, musculoskeletal, etc. It has been reported that girls with T18 have a greater life expectancy than boys [6-9]. Trisomy 13 (T13) or Patau syndrome is the third most common trisomy and the incidence of T13 is 1 in 12,000 to 16,000 live births. Over 95% of these fetuses are lost due to spontaneous abortion [1,5]. Less than 10% of T13 infants survive the first year of life [1]. Patients with T13 exhibit the unusual phenotype of midline facial and central nervous system defects, such as cleft lip and palate, single orbit, and alobar holoprosencephaly, small ears, and malformation. In addition to this, these patients often have congenital heart disease, abnormalities of the liver, kidneys, lungs, and pancreas [1].

Trisomy patients have needed the support of a wide range of medical professionals, since children [10]. The clinical manifestations of these disorders range from relatively innocuous deviations to life-threatening consequences. A trisomy child should have checkup and treatment from several medical professionals, including developmental pediatrician, cardiologist, ophthalmologist, orthopedic specialist, physical and occupational therapist, speech-language therapist, as well as audiologist [11,12].

Currently, prenatal screening via serum screening tests in pregnant woman are included in Thailand’s Universal Health Coverage, which covers approximately 80% of the Thai population, and the Thai government intends to expand the prenatal screening test policy to all pregnant women in the near future [13]. Aside from this, more effective methods of detecting fetal cell-free DNA (cfDNA) in maternal plasma, a process known as non-invasive prenatal testing (NIPT), have been developed in recent years, allowing for the detection of embryonic chromosomal abnormalities [14]. Before enforcing prenatal screening for all pregnant women, governments ought to make sure the tests are affordable. However, prenatal screening for T21 patients has only been evaluated financially in a small number of Thai studies. In analysis of serum screening conducted in 2010, Pattanaphesaj et al used a lifetime cost of T21 to be 65,045 USD [15]. From this premise, in 2017, Oraluck et al employed serum screening and NIPT costs across the lifespan of T21 patients from a study by Pattanaphesaj et al. in their calculations [16]. In contrast to the above studies, economic evaluation of serum screening and NIPT was undertaken by Wanapirak et al., using lifetime cost data from the United States, which was 583,144 USD [17].

Until recently, no study had been explored the lifetime cost of T13, T18, and T21 in Thailand. Consequently, the objective of this study was to estimate the lifetime cost of T13, T18, and T21 at Siriraj Hospital, Thailand’s largest teaching hospital, where a wide range of medical professionals and cutting-edge technology are available to care for patients with these conditions. In order to illustrate and generalize in Thailand, it is necessary to employ up-to-date, locally-relevant lifetime costs in the cost-effectiveness analysis of prenatal screening tests.

**Materials and methods**

**Study design**

This cross-sectional study was performed by a prevalence-based societal perspective approach. A retrospective study was conducted using a hospital electronic database for a one-year time period to calculate direct medical cost associated with T13, T18, and T21 treatment. Direct non-medical cost incurred by patients and their families during treatment, including travel and meal expenses, were calculated from standard costing [18], and indirect cost was determined using a human capital approach.

**Study population**

With the approval of Siriraj Institutional Review Board (SIRB) (MU-MOU COA 657/2021), inpatient and outpatient care data for cost calculation in 2016 were retrieved from the Siriraj Informatics and Data Innovation Center (SiData+), Faculty of Medicine, Siriraj Hospital. Classification of Disease-Clinical Modification, the Tenth Revision codes (ICD-10-CM) was used to identify T13, T18, and T21 as follows: Q90.0, Trisomy 21 without mosaicism (meiotic nondisjunction); Q90.1, Trisomy 21 with mosaicism (mitotic nondisjunction); Q90.2, Trisomy 21 translocation; Q90.9, Down syndrome not otherwise specified; Q91.0, Trisomy 18 without mosaicism (meiotic nondisjunction); Q91.1, Trisomy 18 with mosaicism (mitotic nondisjunction); Q91.2, Trisomy 18 translocation; Q91.3, Trisomy 18 not otherwise specified; Q91.4, Trisomy 13 without mosaicism (meiotic nondisjunction); Q91.5, Trisomy 13 with mosaicism (mitotic nondisjunction); Q91.6, Trisomy 13 translocation; Q91.7, Trisomy 13 not otherwise specified.

**Cost estimation**

***Direct cost***

Direct medical costs were divided into service fee, drug and medical devices, laboratory diagnosis, radiology examination, rehabilitation, operations, and others (dental care, psychology, blood transfusion). We divided patients into three groups based on their age: children (0-14 years), adults (≥15 years), and all patients. Direct non-medical costs, including transportation and meals, were calculated using standard costing [18] multiplied by the average number of outpatient care visit and length of stay for inpatient care per case. Consumer Price Index (CPI) was used to adjust the cost from 2016 to 2022. The costs were also converted from Thai baht (THB) to US dollars (USD) using the exchange rate of 38.08 baht per 1 USD (2022 prices).

***Indirect cost***

Indirect costs, including productivity loss of the patients and the opportunity cost for the caregivers, were calculated using the human capital approach [19,20]. Labor productivity was determined using the average gross domestic product (GDP) per worker in 2021. The Thai GDP per capita (7,233.4 USD) was multiplied by the average survival year of trisomy [21] to calculate productivity loss of caregivers. For productivity loss of trisomy patients, the working age range (45 years) was multiplied by the Thai GDP per capita. Beyond one year, costs increased at a rate of 4% per year and were discounted at a rate of 3% per year. Following that, the costs were converted to its current value using the formula: PV = FV x [1/(1+r)^n^, where PV = present value, FV = future value, r = discount rate, and n = number of survivor year. The estimated lifetime cost of T13 and T18 was based on a one-year survival rate [1], whereas T21 was assumed to be 50 years [2].

**Statistical analysis**

Statistical analysis was performed using the Microsoft Excel 2019 (Microsoft, WA, USA). Demographic data were analyzed using descriptive statistics. Costs were presented as mean with standard deviation (SD) to represent the cost burden and as median with minimum and maximum as well as interquartile range (IQR) to represent the probable anticipated cost for an individual. Statistical differences between T21 with CHD and without CHD were calculated using the nonparametric Mann-Whitney U-test for non-normally distributed data, and the parametric equivalent Student’s t test for normally distributed data. A P-value less than .05 was regarded as being statistically significant.

**Results**

***Demographic characteristics of patients***

Table 1 presents the demographic characteristics of T13, T18, and T21 patients. Data on 371 patients were collected in this study. There were 241 and 124 patients with T21 receiving outpatient and inpatient cares, respectively. The age of the affected T21 ranged from 0 to 59 years, with a mean age of 10.4 ± 9.6 years and a median age of 7 years. For analysis of outpatient and inpatient cares for T13 and T18, there were five and seven patients, respectively. The age of T13 patients ranged from 0 to 14 years, with a mean age of 4.8 ± 5.4 years and a median age of 3 years, while T18 patients ranged in age from 0 to 14 years, with a mean age of 4.4 ± 6.6 years and a median age of 1 years. Approximately 55% of T21 patients were female and 45% were male. T13 and T18 were 80% and 100% female, respectively. Approximately 90% of T18 patients and 60% of T21 patients reported ever having had a cardiac problem. Average outpatient department visits for T13, T18, and T21 patients were 9±9.6, 6±7.5, 14.2±13 per year, respectively. Average duration of inpatient stay 55 ± 109 days for T13 patients, 46 ± 54 for T18 patients, and 88 ± 211 days for T21 patients.

Table 1 Demographic characteristics of trisomy patients

| **Demographic characteristics** | **Outpatient care [N = XX]** | | | **Inpatient care [N = XX]** | | |
| --- | --- | --- | --- | --- | --- | --- |
|  | **Trisomy 13  N (%)** | **Trisomy 18  N (%)** | **Trisomy 21  N (%)** | **Trisomy 13  N (%)** | **Trisomy 18  N (%)** | **Trisomy 21  N (%)** |
| Age, years |  |  |  |  |  |  |
| Mean ± SD | 4.8±5.4 | 4.4±6.6 | 10.4±9.6 | 4.8±5.4 | 4.4±6.6 | 9.9±10.5 |
| Median (IQR) | 3 (3-4) | 1 (0-8) | 7 (4-13) | 3 (3-4) | 1 (0-8) | 7 (4-12) |
| Range | 0-14 | 0-14 | 0-59 | 0-14 | 0-14 | 0-59 |
| Children <14 year | 5 (100) | 7 (100) | 191 (79.3) | 5 (100) | 7 (100) | 106 (85.5) |
| Adult ≥15 year | 0 (0) | 0 (0) | 50 (20.7) | 0 (0) | 0 (0) | 18 (14.5) |
| Sex |  |  |  |  |  |  |
| Female | 4 (80) | 7 (100) | 132 (54.8) | 4 (80) | 7 (100) | 66(53.2) |
| Male | 1(20) | 0 (0) | 109 (45.2) | 1(20) | 0 (0) | 58(46.8) |
| Congenital heart disease |  |  |  |  |  |  |
| Yes | 0 (0) | 6 (85.7) | 127 (52.7) | 0 (0) | 6 (85.7) | 74 (59.7) |
| No | 5 (100) | 1 (14.3) | 114 (47.3) | 5 (100) | 1 (14.3) | 50 (40.3) |
| Number of Visits, N |  |  |  |  |  |  |
| Mean ± SD | 9±9.6 | 6±7.5 | 14.2±13 | 2.4 ± 2.2 | 2.2 ± 1.5 | 1.5 ± 1.4 |
| Median (IQR) | 6 (3-10) | 3(1.5-6.5) | 10 (6-18) | 1 (1-3) | 2 (1.25-2) | 1 (1-2) |
| Range | 1-25 | 1-22 | 1-110 | 1-6 | 1-5 | 1-10 |
| Length of Stay, Days |  |  |  |  |  |  |
| Mean ± SD | NA | NA | NA | 55 ± 109 | 46 ± 54 | 88 ± 211 |
| Median (IQR) | NA | NA | NA | 6 (1-18) | 17 (9 - 90) | 23 (15 – 66) |
| Range | NA | NA | NA | 1 - 248 | 4 - 116 | 3 – 1,900 |

SD; Standard Deviation, IQR; Interquartile Range, NA; Not Applicable

***Cost of illness***

Mean annual direct medical costs for T13, T18, and T21 for outpatient care ranged from 183.2 to 655.2 USD. For inpatient care, mean annual direct medical costs ranged from 2,507-14,790.5 USD. Mean and median costs raised with age (Table 2).

Table 2 Total annual direct medical costs with Trisomy 13, Trisomy 18, Trisomy 21 (USD)

| **Age group** | **Subject** | | **Annual direct medical cost [USD]** | | | | | |
| --- | --- | --- | --- | --- | --- | --- | --- | --- |
|  | **M** | **F** | **Mean** | **SD** | **Median** | **Min** | **Max** | **Interquartile Range (IQR)** |
| **Outpatient care** | | | | | | | |  |
| Trisomy 13 | | | | | | | |  |
| Children = 5 | 1 | 4 | 183 | 176 | 121 | 10 | 432 | 59-294 |
| Trisomy 18 | | | | | | | |  |
| Children = 7 | 0 | 7 | 321 | 379 | 221 | 36 | 1134 | 92-336 |
| Trisomy 21 | | | | | | | |  |
| All age = 241 | 109 | 132 | 655 | 3,887 | 228 | 2 | 60,058 | 130-467 |
| Adult = 50 | 25 | 25 | 1,768 | 8,464 | 231 | 11 | 60,058 | 91-604 |
| Children =191 | 84 | 107 | 364 | 450 | 221 | 2 | 3,519 | 136-424 |
| **Inpatient care** | | | | | | | |  |
| Trisomy 13 | | | | | | | |  |
| Children = 5 | 1 | 4 | 14,791 | 23,821 | 780 | 290 | 55,559 | 727-16,596 |
| Trisomy 18 | | | | | | | |  |
| Children = 7 | 0 | 7 | 3,815 | 5,468 | 1020 | 214 | 14,244 | 824-4,482 |
| Trisomy 21 | | | | | | | |  |
| All age = 124 | 58 | 66 | 2,507 | 4,605 | 919 | 54 | 27,036 | 523-2,201 |
| Adult = 18 | 9 | 9 | 3,278 | 5,037 | 1317 | 54 | 18,427 | 563-3,404 |
| Children = 106 | 49 | 57 | 2,376 | 4,540 | 851 | 70 | 27,036 | 530-2,165 |

*Cost components*

For outpatient care, drug and medical device costs were large contributors of the total direct medical costs for T13 and T21 patients, while laboratory costs were considerable for T18 patients. For inpatient care, drug and medical device costs were the greatest for T13, whereas drug and medical device cost were the highest for T13, while service fee and operation costs were highest for T18 and T21, respectively (Table 3 and 4).

Table 3 Total annual direct medical costs for individuals with Trisomy 13, 18, 21 classified by type of costs (USD)

| Age group | **Service** | | **Drug and medical devices** | | **Laboratory** | | **Radiology** | | **Rehabilitation** | | **Operation** | | **Others** | |
| --- | --- | --- | --- | --- | --- | --- | --- | --- | --- | --- | --- | --- | --- | --- |
|  | **Mean** | **Median** | **Mean** | **Median** | **Mean** | **Median** | **Mean** | **Median** | **Mean** | **Median** | **Mean** | **Median** | **Mean** | **Median** |
|  | **SD** | **(range)**  **(IQR)** | **SD** | **(range)**  **(IQR)** | **SD** | **(range)**  **(IQR)** | **SD** | **(range)**  **(IQR)** | **SD** | **(range)**  **(IQR)** | **SD** | **(range)**  **(IQR)** | **SD** | **(range)**  **(IQR)** |
| **Outpatient care** | | | | | | | | | | | | | | |
| Trisomy 13 | | | | | | | | | | | | | | |
| Children | 23.8 | 16.4 | 146.6 | 55.6 | 27.8 | 30.6 | 32.3 | 32.3 | 40.9 | 40.9 | 26.4 | 26.4 | 73.1 | 73.1 |
|  | 20.5 | (7.1-59.4)  (14.8-21.4) | 203.2 | (4.8-379.4)  (30.2-217.5) | 22.9 | (2.6-47.5)  (11.3-47.1) | 36.4 | (6.6-58.1) (19.5-45.2) | 24.3 | (23.7-58.1) (32.3-49.5) | NA | NA | NA | NA |
| Trisomy 18 | | | | | | | | | | | | | | |
| Children | 17.6 | 14.2 | 69.3 | 53.9 | 178.3 | 147.4 | 46.3 | 23 | 23.7 | 23.7 | 0 | 0 | 44.9 | 21.1 |
|  | 17.5 | (2.4-47.5) (2.5-26.9) | 72.6 | (5.7-206.6) (11-98.5) | 190.8 | (17.8-578.7) (56.7-195.3) | 67.5 | (6.6-166.2) (9.8-26.1) | NA | NA | 0 | 0 | 57.9 | (2.6-110.8) (11.9-66) |
| Trisomy 21 | | | | | | | | | | | | | | |
| All age | 54.5 | 34.2 | 464.4 | 41 | 90.4 | 68.1 | 35.8 | 15.4 | 82.2 | 42.5 | 39.5 | 15.9 | 30.5 | 11.9 |
|  | 63.8 | (2.4-457) (17-66.4) | 4,021.0 | (0.3-59,770.2) (14.2-155.5) | 97.3 | (2.1 - 968.1) (35.4-112.1) | 92.6 | (6.6-962.4) (6.6-29.2) | 117.3 | (2.7-871.9) (21.2-104.8) | 83.6 | (2.7-694.2) (8-31.8) | 79.5 | (3.7-704.4) (5.3-28.7) |
| Adult | 44.8 | 28.00 | 1,708.1 | 76.9 | 105.3 | 63.6 | 61.9 | 26.5 | 95.5 | 35.8 | 43.5 | 15.9 | 89.1 | 11.8 |
|  | 43.5 | (2.4-175.2) (16.7-46.5) | 8,894.7 | (8.3-59,770.2) (19.5-210.3) | 150.8 | (2.1-968.1) (35-116.8) | 181.9 | (6.6-962.4) (6.6-34.8) | 119.0 | (2.7-345) (23.9-152.6) | 67.0 | (2.7-233.3) (8-33.2) | 202.2 | (3.7-704.4) (6.5-604) |
| Children | 57 | 38 | 150 | 35.9 | 86.6 | 68.6 | 30.4 | 13.3 | 80.8 | 42.5 | 38.7 | 15.9 | 21.1 | 11.9 |
|  | 68 | (2.4-457) (17.1-67) | 345.7 | (0.3-2,725.4) (13-129.9) | 78.1 | (2.7-461.3) (36.7-111.5) | 59 | (6.6-527.1) (6.6-26.5) | 117.7 | (5.3-871.9) (21.2-102.2) | 87.1 | (8-694.2) (8-31.8) | 24.7 | (3.7-173.8) (5.3-26.5) |
| **Inpatient care** | | | | | | | | | | | | | | |
| Trisomy 13 | | | | | | | | | | | | | | |
| Children | 4,135.7 | 261.7 | 6,399.2 | 58.2 | 1573.6 | 1050 | 544.8 | 216 | 0 | 0 | 2071.1 | 586.7 | 997.9 | 288.5 |
|  | 6,716.4 | (63.2-15,649.7) (126.1-4,577.9) | 11,976.7 | (13.7-27,560.9) (14.5-4,348.9) | 1880.2 | (13.3-4,181.3) (309.9-2,313.7) | 748.9 | (16.5-1,402) (116.3-808.9) | 0 | 0 | 2749.6 | (35-6,477) (198.7-3,058.1) | 1484.8 | (0.8-2,704.3) (144.7-1,496.4) |
| Trisomy 18 | | | | | | | | | | | | | | |
| Children | 1,430.8 | 861.4 | 225.6 | 59.1 | 287.6 | 140 | 67.3 | 63 | 101.3 | 101.3 | 1162.6 | 37.3 | 888.8 | 77.5 |
|  | 1,642.1 | (38.3-4,363.7) (321.3-1,954.2) | 324.4 | (7.2-808.5) (15.2-329.3) | 373.4 | (2.1-897) (15.6-383.3) | 68.8 | (6.6-136.7) (9-121.3) | NA | NA | 2322.1 | (6.6-5,302.2) (6.8-459.8) | 1943.2 | (3.2-4847.8) (13-270.9) |
| Trisomy 21 | | | | | | | | | | | | | | |
| All age | 713.1 | 232.8 | 315.2 | 30.5 | 285.9 | 59.3 | 156.6 | 39.3 | 81.6 | 94 | 1277.1 | 509.7 | 106.7 | 5 |
|  | 1970.8 | (39.8-16,248) \(90-548.1) | 1046.5 | (0.2-7,936.2) (13.5-141.5) | 742.4 | (1.6-5,366.5) (19.1-220.7) | 311.5 | (4.2-1,795) (13.3-114.4) | 68.8 | (11.9-182.1) (22-98.2) | 1631.9 | (5.3-9,486.7) (357.9-1,588.8) | 317.7 | (0.8-2,320) (1.6-72.2) |
| Adult | 555.2 | 164.6 | 615.4 | 55.5 | 440.1 | 59.3 | 402.4 | 191.2 | 0 | 0 | 1648.2 | 918.1 | 301.6 | 5 |
|  | 956.8 | (39.8-3,302) (63.7-493.7) | 1444.5 | (2.3-4,583.9) (14.3-185.1) | 913.3 | (2.1-3,019.8) (13.9-258.8) | 476.5 | (9.8-1,038) (34.4-799.5) | 0 | 0 | 1996 | (10.6-6,869.4) (416.1-1766.4)) | 762 | (0.8-2,320) (0.8-116.5) |
| Children | 739.9 | 244.7 | 267 | 30.1 | 260.2 | 59.6 | 127.7 | 39.3 | 81.6 | 94 | 1208.9 | 503.6 | 87.7 | 5.3 |
|  | 2096.8 | (44.6-16,248.2) (93.1-555.4) | 968.3 | (0.2-7,936.2) (13.4-138) | 712.5 | (1.6-5,365.5) (20.5-199) | 279 | (4.2-,1795) (13.3-84.9) | 68.8 | (11.9-182.1) (22-98.2) | 1559.9 | (5.3-9,486.7) (345.5-1,588.8) | 236.1 | (0.8-1,832.9) (1.6-68.8) |

SD; Standard Deviation, IQR; Interquartile Range

Table 4 Percentage of direct medical costs for individuals with trisomy 13, 18, 21

| **Age group** | **Service**  **(%)** | **Drug and**  **medical devices**  **(%)** | **Laboratory**  **(%)** | **Radiology**  **(%)** | **Rehabilitation**  **(%)** | **Operation**  **(%)** | **Others**  **(%)** |  |
| --- | --- | --- | --- | --- | --- | --- | --- | --- |
| **Outpatient care** | | | | | | | | |
| Trisomy 13 | | | | | | | | |
| Children = 5 | 13.0 | 48.0 | 12.2 | 7.1 | 8.9 | 2.9 | 8.0 |  |
| Trisomy 18 | | | | | | | | |
| Children = 7 | 5.5 | 21.6 | 55.6 | 10.3 | 1.1 | 0.0 | 6.0 |  |
| Trisomy 21 | | | | | | | | |
| All age = 241 | 8.3 | 65.6 | 13.2 | 3.5 | 5.5 | 2.1 | 1.7 |  |
| Adult = 50 | 2.5 | 86.9 | 5.6 | 1.9 | 1.1 | 0.7 | 1.2 |  |
| Children =191 | 15.7 | 38.4 | 22.9 | 5.6 | 11.2 | 3.9 | 2.3 |  |
| **Inpatient care** | | | | | | | | |
| Trisomy 13 | | | | | | | | |
| Children = 5 | 28.0 | 43.3 | 8.5 | 2.2 | 0.0 | 14.0 | 4.0 |  |
| Trisomy 18 | | | | | | | | |
| Children = 7 | 37.5 | 5.9 | 6.3 | 1.2 | 0.4 | 25.4 | 23.3 |  |
| Trisomy 21 |  |  |  |  |  |  |  |  |
| All age = 124 | 28.4 | 12.5 | 10.3 | 2.9 | 0.1 | 42.3 | 3.5 |  |
| Adult = 18 | 16.9 | 17.7 | 11.9 | 4.1 | 0.0 | 44.7 | 4.6 |  |
| Children = 106 | 31.1 | 11.2 | 9.9 | 2.6 | 0.2 | 41.8 | 3.2 |  |

*Effect of congenital heart disease on costs incurred by trisomy patients*

Total direct medical costs of T21 patients were also compared based on the presence or absence of congenital heart disease (CHD) (Table 5). For outpatient care, only adult group with CHD had significantly higher mean annual direct medical costs than those without CHD. However, all patients and children with CHD receiving inpatient care incurred significantly higher costs.

Table 5 Total annual direct medical costs incurred with an ‘ever’ diagnosis of congenital heart disease (USD) for trisomy 21 patients

| **Age group** | **CHD** | | | | | | | **No CHD** | | | | | | | | **P-value** |
| --- | --- | --- | --- | --- | --- | --- | --- | --- | --- | --- | --- | --- | --- | --- | --- | --- |
|  | **M** | **F** | **Mean** | **SD** | **Median** | **Range** | **IQR** |  | **M** | **F** | **Mean** | **SD** | **Median** | **Range** | **IQR** |  |
| **Outpatient care** | | | | | | | | **Outpatient care** | | | | | | | |  |
| All age [N=127] | 50 | 77 | 923 | 5,324 | 241 | 8 - 60,058 | 134 - 573 | All age [N =114] | 59 | 55 | 357 | 570 | 216 | 2 – 4,962 | 122 - 374 | 0.26 |
| Adult [N = 16] | 7 | 9 | 4,558 | 14,824 | 639 | 48 - 60,058 | 220 - 1,496 | Adult [ N= 34] | 18 | 16 | 455 | 964 | 167 | 11 – 4,962 | 84 - 347 | 0.006 |
| Children [N = 111] | 43 | 68 | 399 | 544 | 221 | 8 - 3,519 | 123 - 480 | Children [N = 180] | 41 | 39 | 315 | 267 | 238 | 2 - 1,514 | 133 - 385 | 0.78 |
| **Inpatient care** | | | | | | | | **Inpatient care** | | | | | | | |  |
| All age [ N= 74] | 31 | 43 | 3,378 | 5,553 | 1,393 | 54 - 27,036 | 659 - 3,444 | All age [N = 50] | 27 | 23 | 1219 | 2111 | 762 | 70 - 13,627 | 467 - 1,171 | 0.0002 |
| Adult [N = 8] | 3 | 5 | 3,475 | 6,172 | 1,360 | 54 - 18,427 | 572 - 2,184 | Adult [N = 10] | 6 | 4 | 3120 | 4271 | 1229 | 434 - 13,627 | 587 - 1,404 | 0.89 |
| Children [N =66] | 28 | 38 | 3,366 | 5,525 | 1,393 | 408 - 27,036 | 659 - 3,444 | Children [N =40] | 21 | 19 | 744 | 481 | 618 | 70 - 1,960 | 445 - 138 | 0.0001 |

CHD; congenital heart disease, SD; Standard Deviation, IQR; Interquartile Range

*Total annual average medical costs incurred by trisomy patients*

Total annual average costs of T13, 18 and 21 are shown in table 6. The proportion of total annual average cost attributable to indirect costs was the greatest for T13 followed by T18, and T21 patients. T13 patients had the highest annual average cost compared to T18 and T21 patients. Due to longer life expectancies of T21 patients, the lifetime cost of T21 was about 1,153,629 USD.

Table 6 Annual average cost of trisomy patient

| **Type of cost** | **Trisomy 13**  **Mean ± SD** | **Trisomy 18**  **Mean ± SD** | **Trisomy 21**  **Mean ± SD** |
| --- | --- | --- | --- |
| **Annual Direct medical cost (USD)** |  |  |  |
| Outpatient care | 183±176 | 321±379 | 655± 3,887 |
| Inpatient care | 14,791± 23,821 | 3,815±5,468 | 2,507±4,605 |
| Annual direct medical cost | 14,974±23,997 | 4,136±5,847 | 3,162± 8,492 |
| **Annual Direct non-medical cost (USD)** |  |  |  |
| Transportation | 561±1,040.0 | 456±539 | 903±1,964 |
| Food | 127±213 | 99±118 | 206±387 |
| Annual Direct non-medical cost | 688±1,253 | 555±658 | 1,109±2,351 |
| **Indirect cost (USD)** |  |  |  |
| Productivity loss of trisomy patient | 7,233 | 7,233 | 7,233 |
| Productivity loss of care giver | 7,233 | 7,233 | 7,233 |
| Annual indirect cost | 14,467 | 14,467 | 14,467 |
| **Annual Total costs (USD)** | 30,128 | 19,158 | 18,738 |
| **Lifetime cost (USD)** | 30,128 | 19,158 | 1,153,629 |

**Discussion**

This study was the first to investigate the lifetime costs incurred by T13, T18, and T21 patients in Thailand. We explored direct medical, direct non-medical, and indirect costs incurred by trisomy patients of all ages in a single study. Typically, either children or adults have been the subjects of cost studies. Our study showed the annual average direct medical costs for children with T13 and T18 were 14,974 and 4,136 USD, respectively, which were lower than a previous study from the US revealing mean annual hospital charges of 30,021 USD and 39,537 USD [26] given the fact that direct healthcare costs incurred in Thai hospitals are much lower compared to those in the US hospitals.

Our study demonstrated that mean annual direct medical costs for T21 children aged 0-14 years (2,740 USD) was much lower compared to the studies in Australia, the US and Asia. In Boulet’s study done in Western Australia the average medical costs for children with Down syndrome aged 0 to 4 years was 36,384 USD and median cost of 11,164 USD per year.[22] Moreover, Dawson AL et al uncovered that around 70% of hospitalizations for children with Down syndrome occurred in the first year of life in the US, leading to an increase in healthcare costs [11]. When compared to non-Down syndrome children in the US, Down syndrome patients under 18 years of age experienced total annual out-of-pocket medical costs of 18,248 USD and third-party payers incurred costs of 230,043 USD [23]. Furthermore, the study by Park et al in Korean demonstrated that the medical costs of children with Down syndrome were almost 10-fold higher than those of children without Down syndrome [24].

In addition, T21 adults aged ≥15 years in our study (5,046 USD) consumed annual average direct medical cost less than that in the US (18,241 USD) based on the Nationwide Inpatient Sample database [25]. The annual mean direct medical cost was much higher among Down syndrome with dementia aged 45–89 (35,011 USD) compared to those without dementia (24,401 USD), due to higher inpatient services care, multimorbidity, and more primary care physician and emergency department visits [26]. However, our study showed similar average direct medical costs among T21 adults to the study conducted in a hospital covered by Taiwan's national health insurance (5,006 USD) [27].

Besides, our study revealed that T21 patients with CHD in adult groups who receiving outpatient care and children who receiving inpatient cares had significantly higher mean annual direct medical costs than those without CHD in all patient groups. This is consistent with Boulet SL et al study in the Western Australia suggesting that the mean and median costs for Down syndrome newborns with CHD were five to seven times more than for Down syndrome infants without CHD [22]. Similarly, mean annual inpatient cost ranged between 9,706 USD and 109,059 USD for Down syndrome with CHD in the US [11].

Furthermore, the average annual direct healthcare costs among Down Syndrome patients aged 0 to 25-year-old in the Western Australia was 4,287 USD, and those costs dropped with age, as hospital, physician, and treatment consumption reduced [10]. In contrast, our study demonstrated that the mean and median costs for T21 raised with age. It was noticed that Down syndrome patients without CHD in all age groups had similar outpatient costs, but adult groups spent more inpatient costs compared to children group. This situation might be due to a difference in costs of health care for T21 children and adult patients. In previous study from Geelhoed, speech therapy and occupational therapy were the most prevalent treatments for T21 children, with mean annual costs of 1,878 USD and 1,505 USD, respectively [10], while the patients were not charged for these treatments in our study. As a result, the greater use of drugs and medical devices by adults compared to children contributed to the rise in adult healthcare costs.

Rendering care for trisomy patient results in increasing direct non-medical and indirect costs for caregivers. Our study estimated direct non-medical cost for trisomy care ranging from 500 to 1,100 USD per year and indirect cost from productivity loss of trisomy patients and caregivers approximately 15,000 USD per year. These costs were lower compared to the study from Stabile and Allin indicating that the family costs for a child with a disability or chronic condition ranged between 20,000 and 60,000 USD per year [28]. Furthermore, our study also found that that a trisomy patient required mostly his or her mother as a caregiver. This is in line with the study of Martınez-Valverde et al in Mexico suggesting that mothers of T21 children had to leave their jobs for caring their children and this might cause a negative effect on family income [12].

Our study revealed the lifetime cost for trisomy patients ranging from 20,000 to 1,200,000 USD depending on the average survival year of the patients, which seemed to be lower compared to the economic costs of childhood disability ranging from 41,000 to 4,300,00 USD according to a systematic analysis [29]. Nevertheless, compared to lifetime economic burden of T21 patients in Asia countries like China (55,000 USD) [30] and Thailand (72,000 USD) [15], our lifetime economic burden of T21 patients was higher.

To the best of our knowledge, this is the first study to demonstrate both direct and indirect costs of patients with T13, T18, and T21 in Thailand. However, this research was limited by the fact that the data were obtained from a single hospital and direct non-medical costs were calculated using standard costing methods. This might result in the underestimation of the cost for trisomy care in Thailand. Notwithstanding these limitations, this research provided not only the evidence of the economic impact of T13, T18, and T21, but also the proper economic assessment for these diseases in Thailand. Future studies should be further investigated on collecting more direct medical cost data from other hospitals and direct non-medical cost data from interviewing trisomy patients and their caregivers.

**Conclusion**

The findings of this study would be useful for determining the lifetime cost of Thai patients with T13, T18, and T21. Total annual average direct medical costs with T13, T18, and T21 ranged from 183.2 USD to 655.2 USD for outpatient care and from 2,507 USD to 14,790.5 USD for inpatient care. Mean and median costs increased with age. The total mean annual direct medical costs were significantly higher in patients with CHD across all patient groups receiving outpatient and inpatient cares. The lifetime costs of T13, T18, and T21 were 30,128 USD, 19,158 USD, and 1,153,629 USD, respectively.

**Acknowledgments**

The authors gratefully acknowledge Assoc. Prof. Panutsaya Tientadakul, Head of the Department of Clinical Pathology, Faculty of Medicine Siriraj Hospital, Mahidol University for her support.

**References**

1.	Levy PA, Marion R. Trisomies. Pediatr Rev. 2018;39(2):104-6.

2.	Gorzoni M.L P, C.F., Ferreira A. Down syndrome in adults: success and challenge. Geriatr Gerontol Aging. 2019;13:111-7.

3.	Asim A, Kumar A, Muthuswamy S, Jain S, Agarwal S. "Down syndrome: an insight of the disease". J Biomed Sci. 2015;22:41.

4.	Capone G, Stephens M, Santoro S, Chicoine B, Bulova P, Peterson M, et al. Co-occurring medical conditions in adults with Down syndrome: A systematic review toward the development of health care guidelines. Part II. Am J Med Genet A. 2020;182(7):1832-45.

5.	Health Quality O. Noninvasive Prenatal Testing for Trisomies 21, 18, and 13, Sex Chromosome Aneuploidies, and Microdeletions: A Health Technology Assessment. Ont Health Technol Assess Ser. 2019;19(4):1-166.

6.	Batees H, Altirkawi KA. Trisomy 18 syndrome: Towards a balanced approach. Sudan J Paediatr. 2014;14(2):76-84.

7.	Cereda A, Carey JC. The trisomy 18 syndrome. Orphanet J Rare Dis. 2012;7:81.

8.	Meyer RE, Liu G, Gilboa SM, Ethen MK, Aylsworth AS, Powell CM, et al. Survival of children with trisomy 13 and trisomy 18: A multi-state population-based study. Am J Med Genet A. 2016;170A(4):825-37.

9.	Wu J, Springett A, Morris JK. Survival of trisomy 18 (Edwards syndrome) and trisomy 13 (Patau Syndrome) in England and Wales: 2004-2011. Am J Med Genet A. 2013;161A(10):2512-8.

10.	Geelhoed EA, Bebbington A, Bower C, Deshpande A, Leonard H. Direct health care costs of children and adolescents with Down syndrome. J Pediatr. 2011;159(4):541-5.

11.	Dawson AL, Cassell CH, Oster ME, Olney RS, Tanner JP, Kirby RS, et al. Hospitalizations and associated costs in a population-based study of children with Down syndrome born in Florida. Birth Defects Res A Clin Mol Teratol. 2014;100(11):826-36.

12.	Martinez-Valverde S, Salinas-Escudero G, Garcia-Delgado C, Garduno-Espinosa J, Moran-Barroso VF, Granados-Garcia V, et al. Out-of-pocket expenditures and care time for children with Down Syndrome: A single-hospital study in Mexico City. PLoS One. 2019;14(1):e0208076.

13.	Free antenatal care (ANC) for pregnant women and partners 2022 [cited 2022 November 17] [Available from: <https://eng.nhso.go.th/view/1/home/News/414/EN-US>.

14.	Badeau M, Lindsay C, Blais J, Nshimyumukiza L, Takwoingi Y, Langlois S, et al. Genomics-based non-invasive prenatal testing for detection of fetal chromosomal aneuploidy in pregnant women. Cochrane Database Syst Rev. 2017;11:CD011767.

15.	Pattanaphesaj J, Tonmukayakul, U., Teerawattananon Y. Cost-benefit Analysis of Prenatal Screening and Diagnosis for Down Syndrome in Thailand. Journal of Health Science 2012;21:667-84.

16.	Oraluck P, Boonsong, O., Wasun, C., Ammarin, T.,Panyu, P. Cost Benefit Analysis of Prenatal Screening Test with Thai NIPT (Thai Non- Invasive Prenatal Test) for Down Syndrome in Developing Countries. Health Care Current Reviews. 2017;5.

17.	Wanapirak C, Buddhawongsa P, Himakalasa W, Sarnwong A, Tongsong T. Fetal Down syndrome screening models for developing countries; Part II: Cost-benefit analysis. BMC Health Serv Res. 2019;19(1):898.

18.	Standard cost list for health technology assessment: Health Intervention and Technology Assessment: HITAP Ministry of Public Health [cited 2022 November 17]. Available from: <https://costingmenu.hitap.net/>.

19.	Lyszczarz B. Indirect costs and incidence of caregivers' short-term absenteeism in Poland, 2006-2016. BMC Public Health. 2019;19(1):598.

20.	Marques N, Gerlier L, Ramos M, Pereira H, Rocha S, Fonseca AC, et al. Patient and caregiver productivity loss and indirect costs associated with cardiovascular events in Portugal. Rev Port Cardiol (Engl Ed). 2021;40(2):109-15.

21.	Thai Gross Domestic Product per capita [cited 2022 November 17]. Available from: <https://data.worldbank.org/indicator/NY.GDP.PCAP.CD?locations=TH>.

22.	Boulet SL, Molinari NA, Grosse SD, Honein MA, Correa-Villasenor A. Health care expenditures for infants and young children with Down syndrome in a privately insured population. J Pediatr. 2008;153(2):241-6.

23.	Kageleiry A, Samuelson D, Duh MS, Lefebvre P, Campbell J, Skotko BG. Out-of-pocket medical costs and third-party healthcare costs for children with Down syndrome. Am J Med Genet A. 2017;173(3):627-37.

24.	Park GW, Kim NE, Choi EK, Yang HJ, Won S, Lee YJ. Estimating Nationwide Prevalence of Live Births with Down Syndrome and Their Medical Expenditures in Korea. J Korean Med Sci. 2019;34(31):e207.

25.	Mendiratta P, Wei JY, Dayama N, Li X. Outcomes for Hospitalized Older Adults with Down Syndrome in the United States. J Alzheimers Dis. 2018;66(1):377-86.

26.	Bayen E, Yaffe K, Cleret de Langavant L, Chen Y, Possin KL. The direct health care cost to Medicare of Down syndrome dementia as compared with Alzheimer's disease among 2015 Californian beneficiaries. Ann Phys Rehabil Med. 2021;64(1):101430.

27.	Hung WJ, Lin LP, Wu CL, Lin JD. Cost of hospitalization and length of stay in people with Down syndrome: evidence from a national hospital discharge claims database. Res Dev Disabil. 2011;32(5):1709-13.

28.	Stabile M, Allin S. The economic costs of childhood disability. Future Child. 2012;22(1):65-96.

29.	Shahat ARS, Greco G. The Economic Costs of Childhood Disability: A Literature Review. Int J Environ Res Public Health. 2021;18(7).

30.	Chen Y, Qian X, Zhang J, Li J, Chu A, Schweitzer SO. Preliminary study into the economic burden of Down syndrome in China. Birth Defects Res A Clin Mol Teratol. 2008;82(1):25-33.
