# Supplementary material for: Economic cost of patients with trisomy 13, 18, and 21 in a tertiary hospital in Thailand
Source: PLoS One. 2023 Nov 16;18(11):e0291918. doi: 10.1371/journal.pone.0291918 (PMC10653468; doi:10.1371/journal.pone.0291918)
Supplement: Attachment — Submitted filename: Response to reviewers-11June23.docx [file pone.0291918.s003.docx]

**Response to Journal requirements & Reviewer:**

**Journal requirements:**

When submitting your revision, we need you to address these additional requirements.

1. Please ensure that your manuscript meets PLOS ONE's style requirements, including those for file naming. The PLOS ONE style templates can be found at

https://journals.plos.org/plosone/s/file?id=wjVg/PLOSOne_formatting_sample_main_body.pdf and

https://journals.plos.org/plosone/s/file?id=ba62/PLOSOne_formatting_sample_title_authors_affiliations.pdf

Response: Thank you very much. We have already checked that our manuscript meets PLOS ONE’s style requirements.

2. Please provide additional details regarding participant consent. In the ethics statement in the Methods and online submission information, please ensure that you have specified (1) whether consent was informed and (2) what type you obtained (for instance, written or verbal, and if verbal, how it was documented and witnessed). If your study included minors, state whether you obtained consent from parents or guardians. If the need for consent was waived by the ethics committee, please include this information.

If you are reporting a retrospective study of medical records or archived samples, please ensure that you have discussed whether all data were fully anonymized before you accessed them and/or whether the IRB or ethics committee waived the requirement for informed consent. If patients provided informed written consent to have data from their medical records used in research, please include this information.

Response: According to your suggestion, we have added the ethics statement under “Study population” sub-heading as follows.

“The ethics approval was granted by the Siriraj Institutional Review Board (SIRB) (MU-MOU COA 657/2021), and the requirement for informed consent was waived by the SIRB committee.” (Study population, line 128-130, page 7)

3. Thank you for stating the following financial disclosure:

“No”

At this time, please address the following queries:

a) Please clarify the sources of funding (financial or material support) for your study. List the grants or organizations that supported your study, including funding received from your institution.

b) State what role the funders took in the study. If the funders had no role in your study, please state: “The funders had no role in study design, data collection and analysis, decision to publish, or preparation of the manuscript.”

c) If any authors received a salary from any of your funders, please state which authors and which funders.

d) If you did not receive any funding for this study, please state: “The authors received no specific funding for this work.”

Please include your amended statements within your cover letter; we will change the online submission form on your behalf.

Response: We would like to provide financial disclosure as follows: “This study receives funding support from the Health Systems Research Institute (HSRI). The funders had no role in study design, data collection and analysis, decision to publish, or preparation of the manuscript." We also include our amended statements within our cover letter.

4. In your Data Availability statement, you have not specified where the minimal data set underlying the results described in your manuscript can be found. PLOS defines a study's minimal data set as the underlying data used to reach the conclusions drawn in the manuscript and any additional data required to replicate the reported study findings in their entirety. All PLOS journals require that the minimal data set be made fully available. For more information about our data policy, please see http://journals.plos.org/plosone/s/data-availability.

Upon re-submitting your revised manuscript, please upload your study’s minimal underlying data set as either Supporting Information files or to a stable, public repository and include the relevant URLs, DOIs, or accession numbers within your revised cover letter. For a list of acceptable repositories, please see http://journals.plos.org/plosone/s/data-availability#loc-recommended-repositories. Any potentially identifying patient information must be fully anonymized.

Important: If there are ethical or legal restrictions to sharing your data publicly, please explain these restrictions in detail. Please see our guidelines for more information on what we consider unacceptable restrictions to publicly sharing data: http://journals.plos.org/plosone/s/data-availability#loc-unacceptable-data-access-restrictions. Note that it is not acceptable for the authors to be the sole named individuals responsible for ensuring data access.

Response: Data cannot be shared publicly because our study obtained data from patients with trisomy 13, 18, and 21 which contained sensitive patient information and there are ethical restrictions on publicly sharing a sensitive data set. Data are available from the Human Research Protection Unit, Faculty of Medicine Siriraj Hospital, Mahidol University (contact via Room 210, 2nd floor, His Majesty the King's 80th Birthday Anniversary 5th December 2007 Building, 2 Wang Lang Road Bangkoknoi, Bangkok 10700 or siethics@mahidol.ac.th) for researchers who meet the criteria for access to confidential data.

**Reviewer #1:**

This study assessed the healthcare use and associated costs for patients with trisomy 13, 18, 21 in Thailand. Overall, the findings of the study benefits to further economic research in Thailand. However, some concerns need to be addressed.

Response: Thank you very much for taking your time to review our manuscript. We feel that our manuscript has been much further improved according to your great suggestions.

1. The title of the study specified “Economic cost of patients with trisomy 13, 18, 21 in

Thailand”. However, only one site with very small sample size (trisomy 13, 18) might not represent the cost for the whole country?

Response: Thank you very much for your great suggestion. We agreed with your suggestion and we have changed the title as “Economic cost of patients with trisomy 13, 18, 21 in a tertiary hospital in Thailand” (Title, line 1, page 1)

2. The study uses descriptive statistics to provide % cost contribution, costs of outpatient

and inpatient. Those costs did not control for other confounders such as age, other co-morbidities, gender, different length of stay. A more robust analysis like GLM regression could provide a more robust result?

Response: Thank you very much for very helpful suggestion. We have performed GLM regression analysis and added more sentences in materials and methods, results, and discussion parts.

“Besides, generalized linear models (GLMs) with gamma distribution were applied to investigate the relationship between annual total direct medical cost and confounding factors i.e., age, female gender, number of outpatient or inpatient visits, and having CHD [22].” (Statistical analysis, line 184-186, page 9)

Table 6 (Results, line 222-232, page 13)

“Moreover, the results of GLM regression analysis indicated that trisomy patients with older age significantly consumed higher direct medical costs in outpatient care.” (Discussion, line 289-291, page 20)

3. The data were retrieved via ICD-10-CM. How many years are there for the database?

Response: Thank you very much for your kind question. One-year data in 2016 were retrieved via ICD-10-CM. We have revised the sentence as follows.

“A hospital electronic database was retrospectively screened to identify patients with T13, T18, and T21 and calculate direct medical cost associated with treatment for a one-year period in 2016.” (line 98-100, page 5)

4. Direct non-medical costs were calculated from standard costing. Why did authors use

the standard costing? Need to explain the strength of the standard costing. Does it still reflect direct non-medical costs nowadays?

Response: Thank you very much for raising this point. We have added the below sentences as suggested.

“The list contains the reference unit cost data of medical services and those incurred by patients receiving treatment in Thailand [18] which are commonly used in cost analysis and better reflect costs nowadays in Thailand.” (Study design, line 105-107, page 5-6).

5. Indirect cost was determined using a human capital approach. The majority of population in this study is children. Is this approach appropriate for those who are not working? In addition, the detail of calculations should be added.

Response: Thank you very much for pointing this out. Indirect costs were calculated using a human capital approach which was applied only adult patients with T21 and caregivers for patients with T13 and T18, as this approach is appropriate for those who are working. We have added the sentence below for clarification.

“According to the Thai HTA guidelines, the human capital approach was applied to calculate indirect costs or productivity loss of both caregivers and adult patients with T21 as well as caregivers for patients with T13 and T18 [19].” (Indirect cost, line 152-154, page 8)

More details of calculations have been added under “Indirect cost” sub-heading. (line 151-166, page 8).

6. Where did 4% (page 6) come from and how did author apply in the formula given?

Response: Thank you very much for raising this point. We have revised the sentences for more explanation as follows.

“To calculate the productivity loss of T21 patients for 35 years, we calculated expected income each year with the increasing rate of 4%, which was obtained from an annual income growth rate during 2012-2022 in Thailand [24]. Based on the recommendation from the Thai HTA guidelines, since cost values are different in different time periods, future values of total expected income or productivity loss during 35 years (FV) should be adjusted to their present values (PV) using an annual discount rate of 3% based on this formula: PV = FV x [1/(1+r)n, where PV = present value, FV = future value, r = discount rate, and n = each year in the future [19].” (Indirect cost, line 169-176, page 8-9)

7. Table 2: what is the unit of annual direct medical cost for OPD and IPD? Is it per visit, per admission, or per patient? Table 1 shows number of visits, and LOS. Authors should classify them by CHD.

Response: Thank you very much for pointing this out. As we have added “Table 1” to demonstrate parameters used for cost calculation (page 9). Therefore, Table 2 is now changed to Table 3 which shows the unit of annual direct medical cost for OPD and IPD per patient. Please see the revised title of Table 3 (page 12). In addition, Table 7 illustrates total annual direct medical costs incurred with an ‘ever’ diagnosis of CHD for trisomy 21 patients as suggested (page 17).

8. Drug and medical device are the main component of costs. Do they include drug costs for co-morbidities?

Response: Yes, drug costs for co-morbidities were included, as cost data of drug and medical devices for all treatment were analyzed in our study. We have revised the sentence to make it clearer as follows.

“Direct medical costs included service fee, drug and medical devices for trisomy treatment and co-morbidities, laboratory diagnosis, radiology examination, rehabilitation, operations, and other services such as dental care, psychology, and blood transfusion.” (Study design, line 95-98, page 5).

9. Table 6 displays the annual average cost. Indirect costs of each trisomy were equal. Why? Furthermore, indirect cost of patient was equal to that of care giver? Patients were children, but care givers were generally their parents.

Response: Thank you very much for raising this point. After carefully checking, we have re-analyzed and found that the indirect costs of T13 and T18 were equal, but those of T21 were different. Thus, we have changed the results in Table 8 (page 18).

For T13 and T18 patients, the indirect costs (productivity loss) were estimated for only caregivers who took care of child patients by the average survival of most patients with T13 and T18 equal to one year multiplied by the Thai Gross Domestic Product (GDP) per capita per year (7,233.4 USD). On the other hand, for T21 patients, the indirect costs were calculated for both caregivers and patients and estimated by the average expected working year of T21 patients (35 years) i.e., average survival years (50 years) minus working age adults at 15 years multiplied by the Thai GDP per capita per year (7,233.4 USD). Even though caregivers were generally their parents who took care of their child patients, they would lose the opportunity to work for gaining income or have productivity loss. Therefore, in this study we applied a human capital approach to calculate the indirect costs or productivity loss of caregivers.

We have added more explanation on how to calculate indirect costs under “Indirect cost” sub-heading (line 152-176, page 8-9).

10. According to Table 1, age range of patient with trisomy 13 and trisomy 18 was 0-14 years. Table 6. Why is lifetime cost equal to annual total cost for Trisomy 13 and Trisomy 18? Patients can live longer than a year.

Response: Thank you very much for your raising this point. We did not use age range of patients with T13 and T18 in our study because it might not reflect the true survival of most patients with T13 and T18. According to published studies on survival of children with T13 and T18, median survival was only 5 and 8 days, respectively. Around 11-17% of T13 newborns and 13% of T18 newborns could survive beyond their first year of life (1). In addition, 10-16% of T13 patients and 10-12% of T18 patients could reach 5 years and only 10% could survive at 10 years (2). There are rare reported cases of these patients surviving into late childhood (3,4). Overall, approximately 90% of these patients would die within one year. Therefore, we applied the survival of one year to calculate the productivity loss of caregivers for these child patients in this study.

References:

1.	Meyer RE, Liu G, Gilboa SM, Ethen MK, Aylsworth AS, Powell CM, et al. Survival of children with trisomy 13 and trisomy 18: A multi-state population-based study. Am J Med Genet A. 2016;170A(4):825-37.

2.	Glinianaia SV, Rankin J, Tan J, Loane M, Garne E, Cavero-Carbonell C, et al. Ten-year survival of children with trisomy 13 or trisomy 18: a multi-registry European cohort study. Arch Dis Child. 2023;108(6):461-7.

3.	Goff RD, Soares BP. Neuroradiological findings of trisomy 13 in a rare long-term survivor. Neuroradiol J. 2018;31(4):412-4.

4.	Khan F, Jafri I. Characterization of a 16-Year-Old Long-Time Survivor of Edwards Syndrome. Cureus. 2021;13(5):e15205.

In addition, how did author calculate lifetime cost? The detail should be added.

Response: We have added more details on lifetime cost calculation at the last paragraph of “Indirect cost” sub-heading (Indirect cost, line 167-176, page 8-9)

11. Authors might display some results using pictures instead of tables.

Response: Thank you very much for your helpful suggestion. We have changed a table of percentage of direct medical costs for individuals with trisomy 13, 18, 21 to figure 1.

**Reviewer #2:**

I thank the editor for the opportunity to review this manuscript. I also commend the authors for undertaking this important work to delineate the cost burden faced by trisomy patients and their caregivers. Overall, the paper is sound and clear with the necessary analyses done. However, I do have concerns with the way the results have been presented in the tables and the discussion section. The discussion section needs to be more concise and clearly link the findings back to the existing evidence. While the authors have attempted this, it seems as though the authors are merely listing other studies rather than to make meaningful linkages back to the findings and how it adds value to the topic/field. I have provided some brief comments below and have added more detailed feedback and edits to the attached document.

Response: Thank you very much for taking your time to review our manuscript. We feel that our manuscript has been much further improved according to your great suggestions.

1. It seems a little confusing that the first sentence says "cross-sectional study" but the second sentence says it is a "retrospective" study. I would think the authors are saying that the electronic database was retrospectively screened to identify patients with T13, T18, T21 and calculate the direct medical costs for a one-year period. If this is right, please amend as suggested.

Response: Thank you very much for your useful suggestion. To avoid the confusion, we deleted "cross-sectional study" and revise the sentence as suggested.

“The inpatient and outpatient care data of patients with T13, T18, and T21 for direct medical cost calculation in 2016 were retrospectively retrieved from the Siriraj Informatics and Data Innovation Center (SiData+), Faculty of Medicine, Siriraj Hospital.” (Study design, line 112-116, page 6)

2. Please elaborate exactly the data fields that were collected to aid in the cost calculation. Maybe including a table of variables that were extracted from the electronic database would be good (unless it is sensitive) to include here so that readers could know what parameters were used to calculate the direct medical costs. You could also include in the table what was used to calculate the indirect costs as well.

Response: We have added “Table 1” showing parameters used to calculate direct medical, direct non-medical, and indirect costs (page 9).

3. I'm aware 15 is the legal age for consent in Thailand. However, may I clarify that children are treated as adults at 15 years old? May I also just clarify the rationale for setting 15 years old as the cut-off and not 18 years, which is the effective age of consent please?

Response: Yes, the legal age for consent is Thailand is 15 years old. We have already added this sentence at “Direct medical and non-medical costs” sub-heading (line 136-139, page 7).

“Even though the effective age of consent in Thailand is 18 years old, we applied 15 years old as the cut-off, since hospitals in Thailand routinely provide care for pediatric patients at age ranging from newborn to 15 years old.”

4. How was patient data handled? Were they all anonymized data? Suggest to include some information about how the extracted data was handled - i.e. were there any personal identifiers, how was the data stored, how long was it stored, was it password-protected, who had access etc. under the methods section. This is important in ensuring responsible use of patient data in studies.

Response: Thank you very much for your great suggestion. We have already added more explanation how patient data were handled in the method section as suggested.

“Patient data from all wards and departments were obtained except trauma department which might not be related to trisomy patients’ co-morbidities. For personal identifiers, coding was used instead of patient’s name and personal data. All raw data were recorded in the principal investigator’s computer with protection code. Principal investigator was the only person who could get access to the data. The data would be stored in the computer until the study has been published. The ethics approval was granted by the Siriraj Institutional Review Board (SIRB) (MU-MOU COA 657/2021), and the requirement for informed consent was waived by the SIRB committee.” (Study population, line 123-130, page 6-7)

6. For all tables, suggest to break them into 2 separate sub-tables for outpatient and inpatient care so that the tables do not look too packed.

Response: Thank you very much for your great suggestion. We have separated a table of total annual direct medical costs for individuals with Trisomy 13, 18, 21 classified by type of costs (USD) for outpatient (Table 4, page 14) and inpatient care (Table 5, page 15) as suggested.

7. Please standardize the number of decimal places/significant figures for p-values. For instance, you can leave it as 2 sig figures. For p-values like this, you can indicate as p <0.01.

Response: Thank you very much. We have changed p-values to “<0.01”. (Table 6, page 16 and Table 17, page 17))

Again, what is the rationale for including gender here? Not really necessary to put in the breakdown of male and female since it doesn't contribute to the cost you are looking at here.

Response: Thank you very much. We have deleted the breakdown of males and females. (Table 17, page 17))

8. Does patient include both outpatients and inpatients? The number does not seem to tally. For example, T21 - 241 outpatients and 124 inpatients = 365 patients in total. For T13 and T18, there is no differentiation between inpatient and outpatients. Is there a reason for this? Suggest to provide the figures separately for inpatients and outpatients for T13 and T18 - what is the number of inpatients with T13 and outpatients with T13 and vice versa for T18 for clarity.

Response: We have checked the figure and the total number of patients was 377 patients. As T13 and T18 patients received both outpatient and inpatient cares, the total number of outpatient and inpatient visits was higher than the number of patients. We have added the sentence below under “Demographic characteristics of patients” sub-heading as suggested.

“Total of 377 patients with T13 (5 patients), T18 (7 patients), and T21 (365 patients) were included in our analysis. All patients with T13 (5 patients) and T18 (7 patients) received both outpatient and inpatient care services, whereas 241 patients with T21 had outpatient visits and 124 received inpatient care.” (line 194-198, page 10)

9. The table of Total annual direct medical costs with Trisomy 13, Trisomy 18, Trisomy 21 (USD) could be better presented - is there a need to show the no. of males and females here? Maybe this could be reflected in the demographics table instead. For this table, it would be good to just focus on outpatient/inpatient and T13, T18, T2. Suggest to maybe switch the columns and rows for the table to be better presented.

Response: Thank you very much for your very helpful suggestion. There is no need to show number of males and females, therefore we deleted it from Table 3 (page 12). We have switched the columns and rows for the table to be better presented as suggested.

**Summary of comments and suggestions from track change on the manuscript**

1. “In order to illustrate and generalize in Thailand, it is necessary to employ up-to-date, locally-relevant lifetime costs in the cost-effectiveness analysis of prenatal screening tests.” This does not sound so smooth. What are you trying to generalize?

Response: Thank you very much. We have already revised the sentence to make it more understandable as follows.

“Our study could provide up-to-date and locally-relevant lifetime costs of patients with T13, T18, and T21 which are necessary to be applied in the cost-effectiveness analysis of prenatal screening tests.” (Introduction, line 87-89, page 5)

2. Can the authors elaborate briefly how the costs were calculated please? Did the family of the patients provide any cost-related information to this study?

Response: Yes, we have already elaborated more how direct non-medical costs were calculated as suggested. The family of the patients did not provide any cost-related information to this study.

“Direct non-medical costs i.e., transportation and meal expenses incurred by patients and their families during outpatient and inpatient visits were calculated by the average number of outpatient care visits and length of stay (LOS) per case analyzed from the hospital electronic database multiplied with the unit costs of transportation per one round trip and three meals obtained from the standard cost list for health technology assessment (HTA).” (Study design, line 100-105, page 5)

3. This answers my previous comment on what constitutes the direct costs but maybe this could be made more clearer in a table rather than mentioning this in the paragraph. You could just say "direct costs were calculated using the parameters as indicated in Table X". You could also include in the table what was used to calculate the indirect costs as well. Just a suggestion for the authors.

Response: We have added “Table 1” showing parameters used to calculate direct medical, direct non-medical, and indirect costs (page 9).

4. Was only transport and meals considered under direct non-medical costs? Were there other variables included? If so, you can provide all these variables in a table as suggested above.

Response: Yes, only transport and meals was considered under direct-non medical costs. We have added “Table 1” showing parameters used to calculate direct medical, direct non-medical, and indirect costs (page 9).

5. “were discounted at a rate of 3% per year.” Please clarify what this means.

Response: Thank you very much for raising this point. We have revised the sentences for more explanation as follows.

“To calculate the productivity loss of T21 patients for 35 years, we calculated expected income each year with the increasing rate of 4%, which was obtained from an annual income growth rate during 2012-2022 in Thailand [24]. Based on the recommendation from the Thai HTA guidelines, since cost values are different in different time periods, future values of total expected income or productivity loss during 35 years (FV) should be adjusted to their present values (PV) using an annual discount rate of 3% based on this formula: PV = FV x [1/(1+r)n, where PV = present value, FV = future value, r = discount rate, and n = each year in the future [19].” (Indirect cost, line 169-176, page 8-9)

6. Does this figure (371 patients) include both outpatients and inpatients? The number does not seem to tally. For example, T21 - 241 outpatients and 124 inpatients = 365 patients in total. For T13 and T18, there is no differentiation between inpatient and outpatients. Is there a reason for this? Suggest to provide the figures separately for inpatients and outpatients for T13 and T18 - what is the number of inpatients with T13 and outpatients with T13 and vice versa for T18 for clarity.

Response: We have checked the figure and the total number of patients was 377 patients. As T13 and T18 patients received both outpatient and inpatient cares, the total number of outpatient and inpatient visits was higher than the number of patients. We have put the number of inpatients with T13 and outpatients with T13 and vice versa for T18 in Table 2 as suggested (page 11).

7. Table 1 add N

Response: N has been added in Table 2 which was Table 1 previously (page 11).

8. “Cost components” Better to include a header here since you are zooming in on the components that made up the total direct medical costs

Response: Thank you very much. We have changed a header to “Total direct medical cost”. (line 210, page 13)

9. “Annual Direct medical cost (USD)” Standardize your headers - either capitalize all the first letters or only the first letter

Response: Thank you very much. We have already corrected this (page 18).

10. At the 2^nd^ paragraph of discussion part, what point is this paragraph trying to make? That the mean annual direct medical costs for T21 is lower? The comparison of costs between the different countries could be better phrased

Response: Yes, we would like to explain that mean annual direct medical costs for T21 patients was lower than other countries. We have revised this paragraph by connecting with the first paragraph, as they are the same points.

“Similarly, mean annual direct medical costs (2,740 USD) in this study was much lower compared to the studies in Australia [24], the US [11, 25]. and Korean [26]. This can be explained that direct healthcare costs incurred in Thai hospitals are much lower compared to those in developed countries.” (Discussion, line 267-270, page 19)

11. “Nevertheless, compared to lifetime economic burden of T21 patients in Asia countries like China (55,000 USD) [30] and Thailand (72,000 USD) [15], our lifetime economic burden of T21 patients was higher.” Maybe better to provide reasons why?

Response: Thank you very much. We have added the reasons as suggested.

“Nevertheless, compared to lifetime economic burden of T21 patients in Asia countries like China (55,000 USD) [33] and Thailand (72,000 USD) [15], our lifetime economic burden of T21 patients was higher for the reason that our direct medical costs data were collected from the largest teaching and tertiary hospital where leading-edge and high cost technology are available to care for these patients and we assumed that T21 patients would receive treatment and have productivity loss for 35 years. Thus, this may overestimate the lifetime cost of T21 patients in our study.” (Discussion, line 312-318, page 21)

12. Last paragraph of discussion part. How does this study contribute to the existing gap?

Response: Thank you very much for great suggestions. We have added the sentences as suggested.

“To the best of our knowledge, this is the first study to demonstrate both direct medical, direct non-medical and indirect costs of patients with T13, T18, and T21 in Thailand which could fill in the gap with the latest lifetime costs of these patients specifically in local Thai context. This information would be necessary to be applied in the cost-effectiveness analysis of prenatal screening tests which can be used for policy decision making whether the tests should be included in the benefit package of the Universal Health Coverage. In addition, the results from this study can help support the Thai government to implement the prenatal screening test policy to all pregnant women in the near future.” (Discussion, line 319-326, page 21)

13. How are the findings important in pushing for more pre-natal screening etc? Link the findings back to address the current gaps.

Response: Thank you very much for great suggestions. We have added the sentences as suggested.

“To the best of our knowledge, this is the first study to demonstrate both direct medical, direct non-medical and indirect costs of patients with T13, T18, and T21 in Thailand which could fill in the gap with the latest lifetime costs of these patients specifically in local Thai context. This information would be necessary to be applied in the cost-effectiveness analysis of prenatal screening tests which can be used for policy decision making whether the tests should be included in the benefit package of the Universal Health Coverage. In addition, the results from this study can help support the Thai government to implement the prenatal screening test policy to all pregnant women in the near future.” (Discussion, line 319-326, page 21)

14. Conclusion, Repetition of findings

Response: Thank you very much. We have revised conclusion as suggested.

“Our study demonstrates direct medical, direct non-medical, indirect costs as well as the lifetime costs of Thai patients with T13, T18, and T21 in a tertiary hospital based on a societal perspective. The results confirm that there is a significant economic burden of patients with T13, T18, and T21 in Thailand. The findings from this study could provide up-to-date direct and indirect cost information for trisomy patients especially in Thai context which would be the best available evidence for conducting economic evaluation of prenatal screening for policy makers 339-345, page 22)
