# Supplementary material for: Economic cost of patients with trisomy 13, 18, and 21 in a tertiary hospital in Thailand
Source: PLoS One. 2023 Nov 16;18(11):e0291918. doi: 10.1371/journal.pone.0291918 (PMC10653468; doi:10.1371/journal.pone.0291918)
Supplement: Attachment — Submitted filename: Response to reviewers-23Aug23.docx [file pone.0291918.s005.docx]

**Response to Journal Requirements:**

Please review your reference list to ensure that it is complete and correct. If you have cited papers that have been retracted, please include the rationale for doing so in the manuscript text, or remove these references and replace them with relevant current references. Any changes to the reference list should be mentioned in the rebuttal letter that accompanies your revised manuscript. If you need to cite a retracted article, indicate the article’s retracted status in the References list and also include a citation and full reference for the retraction notice.

Response: Thank you very much for your suggestion. We have already reviewed the references to ensure that it is complete and correct.

**Response to Reviewer:**

**Reviewer #1:**

Overall, authors have addressed almost all comments. However, some issues need to be clarified.

Response: We would like to thank the reviewers for their helpful comments and suggestions. We feel that the revised paper is much further improved as a consequence of their inputs. Please kindly see our responses to below issues.

Page 8 line 163-166, line 169-171: The estimation of productivity loss was 35 years multiplied by Thai GDP (7233 USD). Is it for caregivers and patients or only caregivers?

Response: The estimation of productivity loss was 35 years multiplied by Thai GDP and this is for T21 patients. We have added more clarification as suggested.

(Indirect cost, line 161-171, page 8)

*“**In this study, we calculated the productivity loss of both caregivers and T21 patients with the average expected survival of 50 years [2], while we considered the productivity loss of only caregivers for T13 and T18 patients who had average survival of only one year according to published studies [1, 21-22]. Therefore, productivity loss of caregivers for T21 patients was estimated by the average expected survival of 50 years, since caregivers have to leave their jobs for caring multiplied by the Thai GDP per capita per year (7,233.4 USD) [23], whereas that of patients with T21 was estimated by the average expected working year of T21 patients (35 years) i.e., average survival years (50 years) minus working age adults at 15 years multiplied by the Thai GDP per capita per year. The summation of productivity loss was divided by 35 years for T21 patients and 50 years for their caregivers to generate an annual productivity loss.”*

Line 164 specified both caregivers and patients. Based on the above estimation, why did productivity loss of caregivers for Trisomy 21 was equal to 9255 USD from Table 8? In addition, productivity loss of patients with trisomy 21 was higher than caregivers who are healthy working people (Table 8)?

Response: Thank you very much for pointing this out. The productivity loss was calculated for both T21 patients and their caregivers. Table 8 presents the annual productivity loss of trisomy patients and their caregivers. The annual productivity loss of caregivers for T21 (9255 USD per year) was calculated by the summation of productivity loss of T21 patients (354,819 USD) divided by 35 years. On the other hand, the productivity loss of T21 patients (9,255 USD per year) was calculated by the summation of productivity loss of caregivers (462,733 USD) divided by 50 years. It is noted that the total productivity loss of caregivers for T21 was higher than that of T21 patients. We have carefully checked our calculation and added the corrected numbers in Table 8 (page 19-20). We have already added more clarification as follows.

(Indirect cost, line 161-181, page 8-9)

*“In this study, we calculated the productivity loss of both caregivers and T21 patients with the average expected survival of 50 years [2], while we considered the productivity loss of only caregivers for T13 and T18 patients who had average survival of only one year according to published studies [1, 21-22]. Therefore, productivity loss of caregivers for T21 patients was estimated by the average expected survival of 50 years, since caregivers have to leave their jobs for caring multiplied by the Thai GDP per capita per year (7,233.4 USD) [23], whereas that of patients with T21 was estimated by the average expected working year of T21 patients (35 years) i.e., average survival years (50 years) minus working age adults at 15 years multiplied by the Thai GDP per capita per year. The summation of productivity loss was divided by 35 years for T21 patients and 50 years for their caregivers to generate an annual productivity loss.*

*To calculate the productivity loss of T21 patients for 35 years and caregivers for 50 years, we calculated expected income each year with the increasing rate of 4%, which was obtained from an annual income growth rate during 2012-2022 in Thailand [24]. Based on the recommendation from the Thai HTA guidelines, since cost values are different in different time periods, future values of total expected income or productivity loss during 35 years (FV) should be adjusted to their present values (PV) using an annual discount rate of 3% based on this formula: PV = FV x [1/(1+r)n, where PV = present value, FV = future value, r = discount rate, and n = each year in the future [19]. In addition, the lifetime costs were calculated by the summation of direct medical costs, direct non-medical costs and indirect costs of caregivers during one year for T13 and T18 patients, 35 years for T21 patients and 50 years for T21 caregivers.”*

**Reviewer #2:**

We would like to thank the reviewers for their helpful comments and suggestions. We feel that the revised paper is much further improved as a consequence of their inputs. Please kindly see our responses to below issues.

1. Do you mean assigning pseudonyms here instead of using the names of patients? If

so, please specify it as such as "coding" here is ambiguous

Response: Thank you very much for your great suggestion. We have changed “coding” to “assigning pseudonyms” as follows.

(Study population, line 126, page 6)

*“For personal identifiers, assigning pseudonyms were used instead of patient’s name and personal data.”*

2. Was it an encrypted device? Please state as "in PI's encrypted computer with

password protection" if so.

Response: Thank you very much for your great suggestion. We have changed to "in PI's encrypted computer with password protection" as suggested.

(Study population, line 127-128, page 6)

*“All raw data were stored in the principal investigator’s encrypted computer with password protection.”*

3. What about the research team members? Were they allowed access to the data at all?

If they were given access to the data for analysis, please state it clearly here.

Response: Thank you very much for your great suggestion. The research team members were not allowed access to the data. We have added the below sentence to make it clearly.

(Study population, line 128-129, page 6-7)

*“Principal investigator was the only person who could get access and analyze the raw data and other researchers could see only summary tables or charts.”*

4. Table 2 Shouldn't this be Adult >=15 instead?

Response: Thank you very much for your great suggestion. We have changed to “Adult >=15” in Table 2 (page 12).

5. I'm not sure what this reference to the Taiwan's national health insurance means here.

Are the authors trying to say it is compared to a "study conducted in a hospital in Taiwan?"

Response: Thank you very much for your great suggestion. Yes, we want to state “a study conducted in Taiwan”. We have changed the sentence as suggested.

(Discussion, line 282-283, page 21)

*“However, our study showed similar average direct medical costs among T21 adults to the study conducted in a hospital in Taiwan (5,006 USD) [30].”*

6. Would it be better to refer to patients who are children as "pediatric" patients?

Response: Thank you for your great suggestion. Yes, we agreed with your suggestion. When we refer to patients who are children, we have changed “children” to “pediatric” patients throughout our manuscript.

7. Please rephrase it to "our study found that caregivers were more likely to be female"

rather than the way it is currently phrased.

Response: Thank you for your great suggestion. We have revised the sentence as suggested.

(Discussion, line 310-311, page 23)

*“Furthermore, our study found that caregivers were more likely to be female.”*
